# The influence of TIVA or inhalation anesthesia with or without intravenous lidocaine on postoperative outcome in colorectal cancer surgery: a study protocol for a prospective clinical study

**DOI:** 10.1186/s13063-022-06157-4

**Published:** 2022-03-18

**Authors:** Alexandru L. Alexa, Tiberiu F. Tat, Daniela Ionescu

**Affiliations:** 1 1st Department of Anesthesia and Intensive Care, Iuliu Haţieganu University of Medicine and Pharmacy, Cluj-Napoca, Romania; 2 Department of Anesthesia and Intensive Care, The Regional Institute of Gastroenterology and Hepatology, “Prof. Dr. Octavian Fodor”, Cluj-Napoca, Romania; 3 Department of Anesthesia and Intensive Care, The Oncology Institute, “Prof Dr. I Chiricuţă”, Cluj-Napoca, Romania; 4 Outcome Research Consortium, Cleveland, USA

**Keywords:** Anesthesia, Colorectal cancer, Propofol, Sevoflurane, Lidocaine

## Abstract

**Background:**

Anesthetic agents are mandatory in colorectal cancer patients undergoing surgery. Studies published so far have shown that anesthetic drugs and intervention may have different impacts on patient’s outcome. Among these drugs, propofol and, more recently, local anesthetics have been mostly targeted.

**Methods/design:**

This study will be a prospective randomized control trial aiming to include 400 patients scheduled for curative colorectal surgery. Patients will be randomized to have general anesthesia with propofol or with sevoflurane. Each study group will be further divided into 2 subgroups of patients, of which one will receive intravenous lidocaine perioperatively. The primary outcome is to compare the incidence of cancer recurrence and survival after propofol versus sevoflurane anesthesia added or not intravenous lidocaine. Secondary outcomes will include the severity of postoperative pain, resumption of bowel function, morphine consumption, length of hospital stay, postoperative chronic pain, and rate of postoperative complications.

**Discussion:**

To our knowledge, this is the first randomized control trial registered on ClinicalTrials.gov designed to compare the effects of two different anesthetic techniques added perioperative intravenous lidocaine infusion on long-term outcomes exclusively in colorectal cancer patients undergoing surgery. The study will bring more accurate data on the effect of propofol-TIVA and perioperative iv lidocaine on the incidence of recurrences after intended curative colorectal surgery.

**Trial registration:**

Clinical Trial Registration NCT02786329. Registered on 1 June 2016

**Supplementary Information:**

The online version contains supplementary material available at 10.1186/s13063-022-06157-4.

## Introduction

In the last years, digestive cancers (liver, colon, and pancreas) increased in incidence and in mortality [1]. Although cancer treatment is multidisciplinary, for digestive cancers, surgical treatment remains the mainstay intervention. Colorectal cancer (CRC) represents a major public health problem as, in 2017, it was ranked as the third dominant cause of death from cancer in the USA [1, 2]. To improve the prognosis of these patients, the impact of a wide range of perianestethic interventions on long-term outcome has been investigated in the recent years [3]. As surgery is the main intervention in CRC, anesthesia and anesthetic interventions are now under investigation as potential ways to influence patient’s response to surgical stress and outcome after surgery. Recently, various experimental and clinical studies found that the immune system in CRC patients may be directly or indirectly influenced during the perioperative period, thus influencing outcome and prognosis [2].

Wigmore’s retrospective clinical study on over 7000 patients found that anesthetic technique may influence long-term outcome and survival when comparing inhalation versus total intravenous anesthesia (TIVA) in different types of cancers [4]. Patients in the inhalation group had a worse outcome regardless of their ASA score, surgical severity, or if they had metastasis at the time of surgery [4]. Wigmore’s study was the starting point for many mostly retrospective clinical studies on this topic. However, even if many studies confirmed Wigmore’s results [5, 6], there are studies reporting no difference between propofol and sevoflurane [7, 8].

More recently, several in vitro and animal studies showed that local anesthetics may have an anti-cancer effect [9–11]. Proposed mechanisms include blockage of sodium channels, alteration of DNA of cancer cells, interference with caspase pathway (caspase-3, Bcl-2, etc.), and others [12]. Local anesthetics may induce DNA fragmentation disrupting the membrane potential of the mitochondria leading to neuron apoptosis [12]. The main mechanism of action of local anesthetics remains the blockage of voltage-gated sodium channels, with their 9 isoforms distributed among various excitable tissues including cancer cells [13].

Lidocaine also has anti-inflammatory effects that may be involved in anti-cancer effects [14, 15]. A recent study showed that perioperative intravenous lidocaine reduced the levels of NETosis and MMP3 [16].

So far, at the date of study registration on ClinicalTrials.gov, no prospective study investigated the effects of local anesthetic associated with TIVA or inhalation anesthesia on long-term outcome in digestive cancers. Our prospective study aims to investigate if there is a difference between TIVA as compared with sevoflurane anesthesia on long-term outcome in colorectal cancer patients undergoing intended radical surgery and if iv lidocaine added to either type of anesthesia has additional effects. Meanwhile, the Vapor-C study was registered and started to recruit patients, but it includes more types of cancers and the completion date will be 2024.

### Objectives

#### Primary outcomes

1. This study aims to compare the influence of TIVA and inhalation anesthesia on long-term outcome in patients with CRC undergoing surgery. Long-term outcomes include the incidence of cancer recurrences and mortality
2. Comparing the incidence of cancer recurrences with lidocaine infusion with placebo

#### Secondary outcomes

1. Overall survival with intravenous lidocaine versus placebo
2. Evaluation of the influence of lidocaine on 24 hours postoperative inflammatory response
3. Evaluation of the influence of lidocaine on the incidence and severity of postoperative pain
4. Monitoring the severity of postoperative pain with verbal response pain score during the first 48 hours postoperatively
5. Evaluation of chronic post-surgical pain with intravenous lidocaine versus placebo
6. Comparison of morphine consumption in the first 24 hours postoperatively
7. Resumption of bowel function
8. Length of hospital stay
9. Rate of 30 days postoperative complications after TIVA versus inhalation anesthesia
10. Rate of 30 days postoperative complications after intravenous lidocaine infusion versus placebo
11. Rate of local anesthetic systemic toxicity incidence

## Patients and methods

After informed consent, an estimated total number of 400 patients scheduled to undergo curative resection of colorectal cancer will be randomized to receive either inhalational anesthesia with sevoflurane or total intravenous anesthesia (TIVA) with propofol, with or without intravenous lidocaine infusion.

### Inclusion criteria

Patients aged 18–80 years, ASA I–III admitted for elective intended curative resection of colorectal cancer under general anesthesia, will be enrolled.

Exclusion criteria include failure to obtain informed consent, age < 18 years or > 80 years, pre-existing chronic pain, chronic medication that may interfere with pain medication (antiepileptics, anti-inflammatory, or corticosteroid medication), contraindication to any medication in the study, significant psychiatric disorders (patients with major depressive disorders, bipolar disorders, schizophrenia, etc.), hepatic dysfunction (ASAT/ALAT > 2 times normal value), renal impairment (serum creatinine > 2 mg/dl), convulsive conditions that required medication in the last 2 years, planned regional analgesia and/or regional anesthesia (epidural or spinal), corticoid-dependent asthma, autoimmune disorders, and antiarrhythmic drugs (amiodarone, verapamil, propafenone) that may interfere with the antiarrhythmic effect of lidocaine.

Dropout criteria include unexpected allergy to one of the used medications, non-curative resection at surgical exploration, intraoperative presence of liver metastasis, patients’ decision to withdraw anytime from the study, and refusal to participate before surgery and at postoperative follow-up.

### Sequence generation and group allocation

**Fig. 1:**
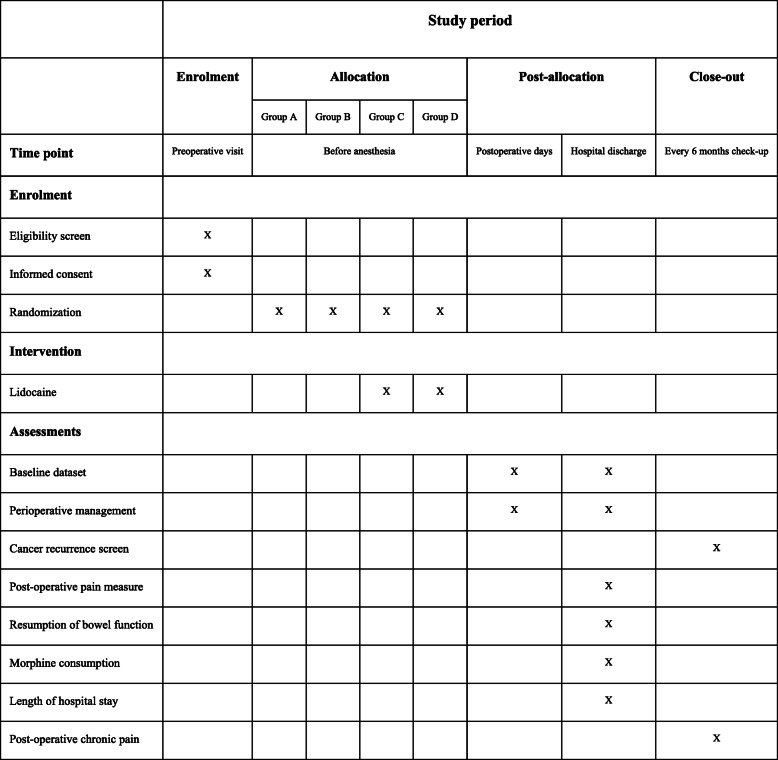
Standard Protocol Items: Recommendations for Interventional Trials (SPIRIT) diagram

Patients will be randomized using a computer-generated random number table into four study groups with approximatively 100 patients each: group A sevoflurane (patients undergoing sevoflurane anesthesia), group B TIVA (patients undergoing total intravenous anesthesia), group C TIVA + lidocaine (patients undergoing total intravenous anesthesia and intravenous lidocaine infusion), and group D sevoflurane + lidocaine (patients undergoing sevoflurane anesthesia and intravenous lidocaine infusion) (Fig. 1). The study statistician will ensure the simple randomization computer-based sequence and will provide unique code numbers for allocation concealment.

### Interventions

The intervention consists in perioperative intravenous infusion with 1% lidocaine to the eligible patients in sevoflurane and TIVA lidocaine groups, respectively. A lidocaine bolus of 1.5 mg/kg will be administered at induction of anesthesia (by using a peripheral catheter). The lidocaine infusion will be maintained during surgery at 2 mg/kg/h at a maximum dose of 200 mg/h. After surgery, the infusion rate will drop to 1–1.5 mg/kg/h (maximum 100 mg/h) of lidocaine and will continue up to 48 hours. Patients receiving this intervention will be admitted in HDU and will be monitored closely after surgery by a study investigator to check for local anesthetic toxicity.

### Perioperative management

Patients enrolled in the study will receive a prophylactic dose of low molecular weight heparin 12 h before surgery. Anesthetic induction will be the same in all 4 groups: fentanyl 2–3 μg/kg, propofol 1.5–2 mg/kg, and atracurium or rocuronium for muscle relaxation at anesthetist discretion (0.5–0.6 mg/kg).

Anesthesia will be maintained in groups A and D with sevoflurane at 1–1.5 minimum alveolar concentration (MAC) increased/decreased in steps of 0.25–0.5 MAC according to bispectral index (BIS) values (40–59).

In groups B and C, anesthesia will be maintained with TCI-propofol (Schnider mode) with an initial effect-site concentration of 4 μg/ml, adjusted in steps of 0.1 μg/ml, to maintain BIS values between 40 and 59.

The patients will be ventilated with a lung-protective regime and a PEEP of 5–6 cm H_2_O, with a fresh gas flow of 2 l/min with a mixture of 50 % oxygen and 50% air.

Intraoperative analgesia will include a multimodal regime with fentanyl in increments of 0.5–1 μg/kg when necessary (blood pressure and/or heart rate increased with over 20% from baseline) and a dose of 1 g of acetaminophen. A bolus of morphine of 0.1–0.15 mg/kg will be administered 30 min prior to extubation. Postoperative analgesia includes intravenous morphine boluses of 0.05 mg/kg and 1 g of acetaminophen every 6 h to maintain an NRS of less than 4 (0 is no pain, 10 is worst pain possible). Morphine 0.025–0.05 mg/kg intravenous boluses will be administered PRN or when NRS will be ≥4 (10-point scale).

### Data collection

Demographic and anesthetic data, as well as surgical data from each enrolled patient, will be registered on a data collection sheet. The study investigators will also collect postoperative follow-up and long-term follow-up data.

Statistical analysis will be performed using the MedCalc Statistical Software version 19.0.7 (MedCalc Software bvba, Ostend, Belgium; https://www.medcalc.org; 2019).

The primary study endpoint will be the incidence of cancer recurrences after TIVA vs sevoflurane anesthesia in colorectal cancer patients undergoing surgery and outcome parameters. Data will be collected from case report forms, anesthetic charts, hospital/ambulatory follow-up visits, and telephone interviews. The appearance of new recurrences will be registered annually.

## Discussion

Studies published so far, mostly retrospective, comparing propofol and sevoflurane for cancer surgery (colon, rectal, breast cancers, etc.) suggested that propofol anesthesia is followed by better overall survival rates than volatile anesthesia for some tumoral types [17, 18]. Similar results were reported by Wigmore et al. in a study that included over 7200 patients undergoing resection for different types of cancer [4]. More specific, propofol-based anesthesia was associated with a higher rate of survival regardless of tumor-node-metastasis stage in colon cancer surgery [19].

Similarly, a retrospective clinical study found that gastric cancer patients undergoing surgery had a better survival rate with TIVA than with volatile anesthetic [20]. Jun and colleagues in patients with esophageal cancer found that patients had better postoperative survival from surgery after TIVA compared with inhalation anesthesia [21].

However, not all studies reported similar results [22–24]. Thus, a retrospective study on lung cancer found no difference in long-term outcome after surgery between inhalation and propofol-based total intravenous anesthesia [8]. No difference in overall and recurrence-free survival was found in over 2700 general anesthesia (TIVA vs inhalation agents) for breast cancer surgeries [24].

Furthermore, there are studies finding a difference in overall survival, but no influence on the recurrence rate [25]. Most recently, Sessler et al. found no difference in the recurrence rate in breast cancer patients undergoing curative surgery when comparing sevoflurane anesthesia (with fentanyl) with paravertebral block and propofol in more than 2000 women [26].

On the other hand, it has been reported that local anesthetics (LAs) used as an adjuvant therapy to multimodal analgesia may have a systemic protective effect against metastasis and tumor growth [27]. The involved mechanisms of action of local anesthetics at the cellular level include the inhibition of proliferation, invasion, and migration [28]. Local anesthetics may also induce apoptosis and change in gene expression through methylation [9]. In clinical concentrations, LAs could be responsible for causing apoptosis, inhibiting proliferation, and migration of cancer cells both in vivo and in vitro models [29].

These effects are produced mainly through voltage-gated sodium channel blockage in cancer cells [30]. The voltage-gated sodium channel (VGSC) blockade seems to be correlated with the tumor invasion and metastasis [3, 27]. Moreover, it appears that lidocaine acts dissimilarly on the microenvironment than on tumor cells [29]. Additionally, Chang and colleagues demonstrated that lidocaine and bupivacaine caused apoptosis and decreased cell viability by inducing caspases 7, 8, 9 in breast cancer cells [31].

It has been shown that colon as well as breast cancer cells express local VGCs, whose inhibition (especially NaV1.5 isoform) attenuates cancer invasion and migration [32]. Previous studies have shown that primary colon cancer cells (SW420) express inferior levels of NaV1.5 than the metastatic cells (SW620) that were isolated from the same patient. It has been demonstrated that the level of NaV1.5 expression is directly proportional to cells’ potential of invasion. An in vitro model of metastatic invasion showed that the migration of SW620 cells through a membrane of Matrigel is reduced, inhibiting the NaV1.5 with lidocaine exposure or by siRNA [11].

To our knowledge, this is the first randomized controlled study to investigate long-term outcomes after elective colon cancer intended curative resection in patients receiving propofol-based anesthesia versus sevoflurane with or without perioperative intravenous lidocaine infusion.

This study has several limitations. Surgery was not done by the same surgeon in all patients, nor the same anesthetist was present for every case. However, the same surgeons with similar expertise were involved in each study group and the perioperative study protocol was strictly followed by every anesthetist. Nonsteroidal anti-inflammatory drugs have been administered at the surgeon’s discretion after lidocaine infusion protocol was complete, when there was the case. Another limitation is that this is a two-institution study with a relatively low number of patients planned to be enrolled. However, patient’s number was estimated based on the results reported by recent retrospective studies and is comparable with the number of patients enrolled in similar studies [25, 33–35].

In conclusion, this prospective randomized control trial will investigate the effects of two types of general anesthesia added or not intravenous lidocaine infusion in patients scheduled for elective colon cancer surgery. The study will be able to provide information on comparative effects of propofol (TIVA) vs sevoflurane on the recurrence rate and long-term outcomes in colorectal cancer patients undergoing intended curative resection and on the effects of lidocaine infusion added to propofol and sevoflurane anesthesia.

## Supplementary Information

**Additional file 1.** SPIRIT 2013 checklist.

## Data Availability

The study database of the present study is available from the corresponding author.

## Abbreviations

CRC: Colorectal cancer
TIVA: Total intravenous anesthesia
ASA: American Society of Anesthesia
DNA: Deoxyribonucleic acid
ASAT: Aspartate aminotransferase
ALAT: Alanine aminotransferase
IV: Intravenous
HDU: High dependency unit
MAC: Minimum alveolar concentration
BIS: Bispectral index
TCI: Target controlled infusion
PEEP: Positive end-expiratory pressure
PRN: Pro re nata
NRS: Numeric rating scale
LAs: Local anesthetics
VGSCs: Voltage-gated sodium channels
siRNA: Small interfering ribonucleic acid
Vapor-C: Volatile Anaesthesia and Perioperative Outcomes Related to Cancer
